# Effects of a Novel Glucokinase Activator, Dorzagliatin, on Glycemic Control and Glucose Fluctuation in Drug-Naïve Patients with Type 2 Diabetes Mellitus

**DOI:** 10.1155/2023/4996057

**Published:** 2023-12-27

**Authors:** Yuming Wang, Xiaofei Su, Wenli Zhang, Yunting Zhou, Xiao Zhou, Wei Yang, Huiqin Li, Jianhua Ma

**Affiliations:** ^1^Department of Geriatrics, Nanjing Drum Tower Hospital, The Affiliated Hospital of Nanjing University Medical School, Nanjing, Jiangsu 210008, China; ^2^Department of Endocrinology, Nanjing First Hospital, Nanjing Medical University, Nanjing, Jiangsu 210000, China; ^3^Department of Pharmacy, Lai'an County People's Hospital, Chuzhou, Anhui 239200, China

## Abstract

**Aim:**

The prevalence rate of type 2 diabetes mellitus (T2DM) has been increasing and a large proportion of patients still do not achieve adequate or sustainable glycemic control on the basis of previous hypoglycemic treatment. In this present study, we explored whether dorzagliatin, a novel glucokinase activator (GKA), could improve glycemic control and lessen glucose fluctuation in drug-naïve patients with T2DM.

**Methods:**

A self-comparative observational study of 25 drug-naïve patients with T2DM (aged 18–75 years and HbA1c of 7.5%–11.0%) treated with dorzagliatin 75 mg twice daily for 52 weeks. Before and after dorzagliatin intervention, the serum levels of hemoglobin A1c (HbA1c), insulin, and C-peptide were measured repeatedly during fasting and after a mixed meal. The continuous glucose monitoring (CGM) device was also used to obtain 24-hour glucose profiles and assess the changes in glycemic variability parameters.

**Results:**

After 52 weeks of treatment with dorzagliatin, a numerally greater reduction in HbA1c of 1.03% from the baseline was observed in patients with T2DM, accompanied by significant improvement in insulin resistance and insulin secretion. Moreover, the standard deviation of blood glucose (SDBG) and the mean amplitude of glycemic excursion (MAGE) derived from CGM data were significantly decreased after dorzagliatin therapy. The 24-h glucose variation profile showed that study patients had obviously lower mean glucose levels during the postprandial period from the baseline to week 52, an effect also demonstrated by the significant decrease in the incremental area under glucose concentration versus time curve for 2 h (iAUC0–2 h) after meals.

**Conclusions:**

This study suggests that dorzagliatin therapy could effectively improve glycemic control and glucose fluctuation in drug-naïve patients with T2DM.

## 1. Introduction

Type 2 diabetes mellitus (T2DM) is a chronic, complex, and multifactorial disease that is characterized by a combination of insulin resistance and *β*-cell dysfunction. The prevalence rate of T2DM has been increasing, year by year, which bring a huge burden for patients, clinicians, and healthcare systems [1]. Several antidiabetic drugs, including metformin, sulfonylureas, and thiazolidinediones, have been approved for the treatment of T2DM [2]. However, these drugs, when used alone or in combinations of multiple agents, have been unable to prevent the deteriorating function of pancreatic islets, and a large proportion of patients still do not achieve adequate or sustainable glycemic control [3, 4]. Therefore, it is imperative that we explore new therapeutic drugs with alternative mechanisms of action that could not only improve glycemic control but also rescue or preserve the function of existing *β*-cells.

Several novel medications that target more recently explored pathogenic pathways related to the gut, brain, and kidneys have been introduced in recent years. Among them, glucagon-like peptide-1 receptor agonists (GLP1-RAs) and sodium-glucose cotransporter-2 inhibitors (SGLT-2is) are widely developed and used all over the world [5, 6]. However, currently available classes of glucose-lowering medications, excepting metformin, have not been directly or effectively targeted with regards to the inhibiting hepatic glucose output, which is closely related to the development of T2DM [7]. Introduction of activators of specific enzyme members of the hexokinase family, glucokinase (GK) activators, has fulfilled this urgent need.

GK is a key enzyme in glycolysis that catalyzes the phosphorylation of glucose to glucose-6-phosphate, acting as a substrate for glycogen synthesis and activating glycogen synthase [8, 9]. It is not widely expressed in pancreatic *β*-cells or hepatic cells but expressed in enteroendocrine cells, neurons, and cells in the anterior pituitary [10]. GK could exert its critical role in maintaining glucose homeostasis, primarily by eliciting glucose-stimulated insulin secretion in pancreatic *β*-cells and promoting glucose uptake and glycogen synthesis in hepatic cells [11, 12]. The important role of GK in maintaining glucose homeostasis has also been proven in patients with GK gene mutations, which can cause many types of diabetes, including maturity-onset diabetes of the young (MODY2) and permanent neonatal diabetes mellitus (PNDM) [13–16]. Thus, GK has become an appealing target for diabetes therapy. Dorzagliatin is currently in clinical trials for the treatment of T2DM due to its novel dual GK activation in the pancreas and liver [17], and preclinical studies have shown that dorzagliatin has a good pharmacokinetic profile in healthy individuals [18] and good efficacy and safety in the treatment of T2DM [17]. However, the effects of dorzagliatin on blood glucose variation in diabetic patients are still unclear. High glucose variability is a risk factor for adverse outcomes in diabetic patients. Several studies have found that glucose variation indicators, such as the standard deviation of blood glucose (SDBG) and mean amplitude of glycemic excursion (MAGE), were associated with chronic diabetes complications [19–21].

In the study reported here, we performed 2-time 3-day continuous glucose monitoring (CGM) at the baseline and the endpoint of dorzagliatin therapy in drug-naïve patients with T2DM, aiming to evaluate the effects of dorzagliatin on long-term glycemic control (52 weeks) and daily glucose fluctuations.

## 2. Materials and Methods

### 2.1. Patient Population

This self-comparative, observational, and single-center study was conducted in the Nanjing First Hospital, Medical University, China, and was approved by the Ethics Committee of the hospital. All procedures were performed in accordance with the principles of Good Clinical Practice, the Declaration of Helsinki, and the relevant Chinese regulations. Written informed consent was obtained from all subjects before study initiation.

Eligible patients were required to meet the following criteria: (1) men or nonfertile women with T2DM diagnosed using the criteria of the World Health Organization in 1999; (2) aged 18–75 years; (3) had received only dietary and exercise interventions at least three months before screening; (4) had exhibited poor glycemic control with hemoglobin A1c (HbA1c) between 7.5% and 11.0%; (5) had a body mass index (BMI) range of 18.5–35.0 kg/m^2^; and (6) agreed to maintain the same dietary and exercise habits throughout the entire research process. Critical exclusion criteria were as follows: (1) clinical diagnosis of type 1 diabetes, diabetes secondary to pancreatic injury, or other special types of diabetes; (2) unexplained severe hypoglycemia or frequent hypoglycemia within 3 months prior to screening; (3) fasting C-peptide <1.0 ng/ml at screening; (4) history of diabetic ketoacidosis, diabetic lactic acidosis, or hyperosmolar hyperglycemic nonketotic coma; (5) uncontrolled hypertension blood pressure (systolic blood pressure >160 mmHg or diastolic blood pressure >100 mmHg) or added/changed antihypertensive drugs or adjusted antihypertensive drug doses within the 4 weeks of screening; (6) history of cardiovascular diseases, malignant tumors, or organ transplantation; (7) triglyceride (TG) >5.7 mmol/L or who took oral lipid-lowering drugs; (8) severe impaired liver and renal function; and (9) history of psychiatric disease or misuse of alcohol or drugs.

### 2.2. Study Design

**Figure 1:**
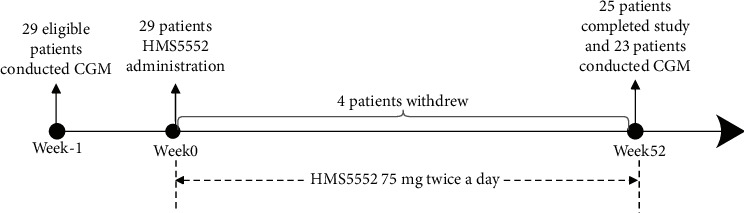
Flowchart.

A total of 29 patients with T2DM who only received dietary and exercise intervention were enrolled in this study. Four patients withdrew consent, and 25 patients completed the study. Eligible patients were treated with dorzagliatin for 52 weeks. Based on previous results which confirmed that 75 mg dorzagliatin twice a day was the optimum effective dose and well tolerated in patients with T2DM, all subjects of this present study were administered dorzagliatin 75 mg daily before breakfast and dinner and there was no change in the dosage of trial medication. A CGM device (iPro2, Medtronic, USA) was used for the baseline and post-treatment assessments, and 23 patients eventually completed the 2-time 3-day CGM. The study design and flowchart of patients are shown in Figure 1.

### 2.3. Clinical and Laboratory Examination

Patient general information and clinical data were recorded during screening for eligibility. Height and body weight were measured using a digital scale, and BMI was calculated as weight divided by height squared (kg/m^2^). Blood samples from all the patients were collected after overnight fasting (≥10 h). Biochemical parameters, including HGB, ALT, AST, total cholesterol (TC), TG, high-density lipoprotein cholesterol (HDL-C), low-density lipoprotein cholesterol (LDL-C), and serum creatinine (SCr), were measured using routine laboratory methods with a HITACHI 7600 device (HITACHI, Tokyo, Japan). The eGFR was calculated using the modification of diet in renal disease equation, i.e., eGFR (mL/min/1.73 m^2^) = 186 × (SCr/88.4) − 1.154 × (age) − 0.203 × (0.742 if female).

Mixed-meal tolerance tests (MMTTs) were conducted at week 0, week 24, and week 52 to assess pancreatic *β*-cell function and insulin resistance. The meal was standardized by China National Cereals, Oils, and Foodstuffs Corporation and contained 353 kcal, 75 g carbohydrate, 1.48 g fat, and 8.0 g protein. The blood samples collected 0 and 120 min after test-meal ingestion were subjected to measurements of insulin and C-peptide (SRL Inc., Tokyo, Japan). The homeostasis model assessment for insulin resistance (HOMA-IR), insulin secretion (HOMA-IS), and *β* cell function (HOMA-*β*) was calculated using previously published procedures [22, 23]. The insulinogenic index, the ratio of the increment in insulin concentration to the increment in glucose concentration (ΔI120/ΔG120), and a derived assessment parameter, the increment in C-peptide concentration to the increment in glucose concentration (ΔC120/ΔG120), were calculated during MMTT, which has been proposed as a measure of insulin secretion [24, 25].

### 2.4. The CGM System

For glucose fluctuation measurements, the CGM system was applied to all subjects before and at week 52 of the treatment. During the CGM monitoring period, all subjects were required to record their meal times and measure their finger peripheral blood glucose at least four times daily to calibrate the CGM system. Data from 0:00 to 24:00 of the second day were used for calculating the following parameters: 24-h mean blood glucose (MBG), SDBG, MAGE, largest amplitude of glycemic excursion (LAGE), average daily risk range (ADRR), low blood glucose index (LBGI), high blood glucose index (HBGI), time in range (TIR, glucose level 3.9–10.0 mmol/L), time above range (TAR, glucose level >10.0 mmol/L), and time below range (TBR, glucose level <3.9 mmol/L). The incremental area under glucose concentration versus time curve from 0 to 2 h (iAUC0–2 h) after three meals was also calculated using the trapezoid rule.

### 2.5. Statistical Analysis

All statistical analyses were performed using SPSS software (version 22.0; SPSS, Inc., Chicago, IL). The Shapiro–Wilk test was used to assess the distribution of data. Changes from the baseline were analyzed using a paired sample *t* test or the signed rank-sum test. *P* values were two-tailed with a significance level of 5%.

## 3. Results

### 3.1. Clinical Characteristics of the Patients

A total of 25 patients with T2DM successfully completed this study. The mean age of the subjects was 58.6 years old (ranges from 47 to 68 years old, 36% women, and mean diabetes duration of 1.44 years). The mean baseline weight and BMI of the participants were 69.23 kg (ranges from 54.0 to 85.5 kg) and 25.50 kg/m^2^ (ranges from 22.02 to 27.73 kg/m^2^), respectively. The clinical characteristics of the patients at the baseline and after 52 weeks of intervention are described in Table 1. ALT and AST levels showed minor increases after dorzagliatin treatment but all remained within the normal range. Total cholesterol and triglyceride levels were also slightly elevated from baseline to week 52. No noteworthy differences were observed in other parameters, such as weight, BMI, SBP, and eGFR.

### 3.2. Glycemic Control and Homeostasis Model Assessment

**Figure 2:**
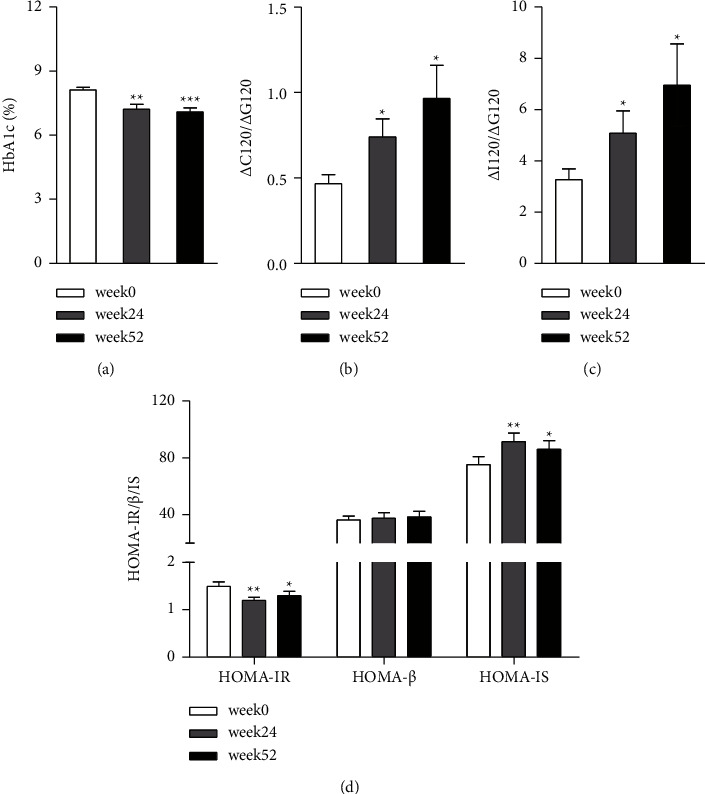
Changes from week 0 to week 52 in HbA1c (a), C_120_/G_120_ (b), I_120_/G_120_ (c), and HOMA-IR/*β*/IS (d), with values presented as the mean. ^*∗*^*P* < 0.05, ^*∗∗*^*P* < 0.01, and ^*∗∗∗*^*P* < 0.001 vs. the baseline. Error bars show SEM.

The most important glycemic control parameter, HbA1c, was measured and compared to baseline levels. Dorzagliatin treatment led to a significant decrease in HbA1c from week 0 to week 24 and week 52 (8.13 ± 0.55% vs. 7.22 ± 0.22%, *P*=0.001, and 8.13 ± 0.55% vs. 7.10 ± 0.89%, *P* < 0.001) (Figure 2(a)). The mean HbA1c change from baseline was −0.91% to week 24 and −1.03% to week 52. The HbA1c response rate (defined as the proportion of patients with HbA1c <7.0% or HbA1c reduction ≥0.6%) was 68.0% at week 24 and 72.0% at week 52.

Pancreatic *β*-cell function and insulin resistance were assessed at week 24 and week 52. The C-peptide-derived dynamic parameter ΔC120/ΔG120 was found to have increased by 0.28 (0.46 ± 0.26 vs. 0.74 ± 0.51, *P*=0.022) and 0.51 (0.46 ± 0.26 vs. 0.97 ± 0.93, *P*=0.013) from baseline to week 24 and week 52 after treatment, respectively (Figure 2(b)). Correspondingly, ΔI120/ΔG120, the insulin-derived dynamic parameter, was also elevated by 1.81 (3.28 ± 1.99 vs. 5.09 ± 4.16, *P*=0.044) and 3.67 (3.28 ± 1.99 vs. 6.95 ± 3.71, *P*=0.026) (Figure 2(c)). In addition, dorzagliatin treatment significantly reduced HOMA-IR from baseline to week 24 and week 52 (1.49 ± 0.49 vs. 1.20 ± 0.35, *P*=0.001 and 1.49 ± 0.49 vs. 1.30 ± 0.46, *P*=0.013), accompanied by increases in HOMA-IS. No significant difference in HOMA-*β* levels was observed between baseline and week 24 or 52 after treatment (Figure 2(d)).

### 3.3. Comparison of 24-h Glucose Variation Profiles

Table 2 showed the glucose fluctuation parameters measured using CGM during dorzagliatin administration. The MBG was significantly reduced after dorzagliatin intervention (10.21 ± 0.45 mmol/L vs. 8.52 ± 0.38 mmol/L, *P*=0.002). Correspondingly, there was a significant decrease in TAR (33.16 (19.10, 77.60) vs. 9.72 (0, 41.93)%, *P*=0.004) and HBGI (9.08 (4.81, 15.09) vs. 4.66 (1.61, 8.47), *P*=0.002) accompanied by an increase in TIR (66.84 (22.40, 80.90) vs. 89.58 (58.07, 98.96) %, *P*=0.005). There were no differences in TBR and LBGI. The parameters of glycemic variability, including SDBG, MAGE, LAGE, and ADRR, were significantly lower than those determined before dorzagliatin treatment.

**Figure 3:**
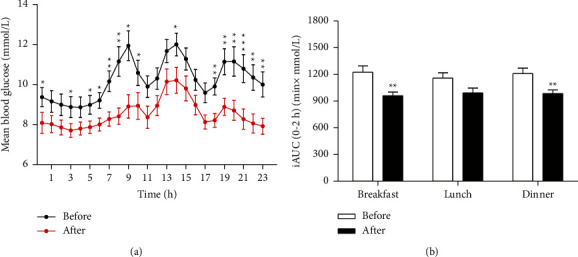
The 24-h glucose variation profiles (a) and the incremental area under glucose concentration versus time curve from 0 to 2 h (iAUC0–2 h) after breakfast, lunch, and dinner (b) of the patients before and after HMS5552 administration, with values presented as the mean. ^*∗*^*P* < 0.05, ^*∗∗*^*P* < 0.01, and ^*∗∗∗*^*P* < 0.001. Error bars show SEM.

The 24-h glucose variation profiles plotted as average blood glucose concentration per hour, measured using CGM in patients after dorzagliatin administration, are shown in Figure 3(a). The incremental postprandial area under the glucose concentration versus time curve from 0 to 2 h (iAUC0–2 h) of the patients after breakfast and dinner significantly declined after treatment (Figure 3(b)).

### 3.4. Safety and Tolerance

25 patients received dorzagliatin treatment and were included in the safety analysis (Table 3). No deaths or drug-related serious adverse events were reported. Upper respiratory tract infection occurred in two patients, and the investigators considered them unrelated to the drug. Hypoglycemia occurred in two (8%) of the 25 patients, both were transient, and no cases of severe hypoglycemia that required medical assistance were reported. We did not observe clinically significant abnormal trends including abnormal hepatic and kidney function. There were no significant differences in body weight or BMI before and after dorzagliatin treatment (Table 1). Regarding blood lipids, total cholesterol, low-density lipoprotein cholesterol (LDL-C), and high-density lipoprotein cholesterol (HDL-C) were within the normal range. The triglyceride level showed a minor increase at week 52, and the incidence of hyperlipidemia was 52% at pretreatment and 60% in the 52 weeks of treatment. One case of hyperlipidemia (TG >5.7 mmol/L) occurred at week 8 and decreased after one month of dietary control without stopping study medication.

## 4. Discussion

One of the aims of this study was to evaluate the effect of dorzagliatin on glycemic control in patients with T2DM. We found that dorzagliatin administration could significantly reduce HbA1c and improve insulin resistance and *β*-cell function at week 24 and week 52. Moreover, we further explored the effect of dorzagliatin on glycemic fluctuation in diabetic patients using the CGM system. Analysis of the CGM data showed that dorzagliatin could not only correspondingly improve glycemic control indicators, such as MBG, TAR, and TIR, but could also lessen glucose fluctuations, especially during the postprandial period.

GK is essential to the pathogenesis of T2DM, and there is evidence that GK activity is reduced by approximately 50% in T2DM patients compared with healthy subjects [26]. The hypoglycemic effect of GK activators was first demonstrated in a 2003 study performed in an animal model of type 2 diabetes [27]. A variety of synthetic or natural GK activators have been reported since 2003, and the efficacy of some of these with regards to the antihyperglycemic activity has been explained in clinical research, including dual activators, such as piragliatin, MK-0941 and AZD1656, and hepatoselective activators, such as TTP-339, GKM-001, TMG-123, and PF-04937319 [27–32]. However, most of these studies were stopped clinical trials because of the incidence of hypoglycemia or other side effects. Dorzagliatin, a structurally novel amino acid-based chemical scaffold that is a dual-acting activator in the pancreas and liver, has been proven to have a hypoglycemic effect in patients with T2DM in a 12-week, phase II clinical trial [17]. Consistent with the results of this phase II clinical study, our results show that dorzagliatin treatment led to significant reductions in HbA1c in drug-naïve patients with T2DM, −0.91% at week 24 and −1.03% at week 52, compared to baseline levels. Furthermore, HbA1c levels were significantly lower throughout the entire study period from week 4 onwards, the first visit of this study. This result was more optimistic than those reported by previous GK activator-related studies. For instance, a phase II trial conducted in Japan found that no significant decrease in HbA1c at week 4 after AZD1656 treatment [30]; a similar result also obtained in the clinical trial of MK-0941 [28]. In addition, no patient experienced serious drug-related side effects or hypoglycemia, weight gain, or abnormal increases in liver enzymes. Our results demonstrate the potential of dorzagliatin to maintain safe use and long-term durability of efficacy even in early diabetes.

HbA1c has been the gold standard for evaluating glycemic control in patients with T2DM and the focus indicator of most recent hypoglycemic therapies. The importance of glucose variability in the long-term management of T2DM is recognized by an increasing number of researchers [33]. Glycemic fluctuations, reflecting short-term oscillations in blood glucose levels, have been proven to play a critical role in the development of chronic diabetes complications, including diabetic neuropathy and diabetic microvascular and macrovascular disease, even in the population of patients who have achieved HbA1c targets [19–21]. CGM is the optimal tool for evaluating glucose variability and hypoglycemia, particularly nocturnal hypoglycemia or asymptomatic hypoglycemia [34]. There were a number of clinical studies to evaluate the effects of drug interventions on glycemic and glucose variability in the population of patients with T2DM by using CGM, such as dipeptidyl peptidase-4 inhibitors (DPP-4is), SGLT-2i, and GLP-1RA [35–38]. However, our study is the first to assess the effect of dorzagliatin on glycemic fluctuation in drug-naïve patients with T2DM.

In the present study, we found that glucose variability indicators such as SDBG, MAGE, LAGE, and ADRR were all significantly reduced at the end of the trial, which was also reflected in the more stable 24-h glucose variation profiles. Glycemic fluctuations mainly include hypoglycemic events and postprandial hyperglycemia [39]. The CGM data obtained from all of the study patients showed only one hypoglycemic event, but no symptoms after dorzagliatin intervention and no differences in terms of TBR and LBGI. There was evidence that postprandial hyperglycemia was associated with the development of cardiovascular disease in patients with T2DM [40], independent of other cardiovascular risk factors and measures of hyperglycemia [41]. Therefore, we further explored changes in incremental postprandial area under the glucose concentration versus time curve from 0 to 2 h (iAUC0–2 h) after three meals; and our results showed that dorzagliatin notably reduced iAUC0–2 h, especially when breakfast and dinner coincided with the time at which medicine was taken. The results indicate reduced blood glucose levels in patients treated with dorzagliatin, reduction that occurs mainly during the postprandial phase, especially after breakfast and dinner, and that contributed greatly to the lower MBG detected and improved glucose fluctuations. In addition, our results demonstrate that dorzagliatin can improve glucose sensitivity and promote insulin secretion in patients with T2DM. Improvements in HOMA-IR and HOMA-IS continued to be seen at week 52 after dorzagliatin treatment, suggesting that dorzagliatin has long-lasting effects on the detrimental symptoms of T2DM. We observed no statistically significant rise in HOMA-*β*; however, ΔI120/ΔG120 and ΔC120/ΔG120, the other two comprehensive *β*-cell function parameters that could reflect postprandial release of insulin and C-peptide [42], were markedly increased after dorzagliatin intervention.

In addition, dorzagliatin treatment did not result in any significant changes in systolic or diastolic blood pressure, body weight, or BMI. In addition, the laboratory parameters related to the liver and kidney function were below the upper limit of normal throughout the study period. Although TC was elevated at the end of the study, primarily with regards to HDL-C, all related measurements were also within normal ranges. Triglyceride levels showed minor but stable increases from baseline to week 52, contradicting results from a previous 12-week phase II clinical trial [1]. But no case of a hyperlipidemia adverse event in the dorzagliatin group was judged by investigators to be related to dorzagliatin throughout the entire study period. Therefore, dorzagliatin demonstrates good tolerability and safety until 52 weeks of treatment.

Recent research has found that the GKA could promote hepatic fat accumulation in db/db mice, accompanied by some dynamic changes in the expression of hepatic genes involved in lipogenesis and gluconeogenesis [43]. But there are currently no reports on the effect of this novel GKA, dorzagliatin, on lipoprotein lipase, hepatic triglyceride content, and hepatic triglyceride synthesis in type 2 diabetes patients. This might be achieved through magnetic resonance imaging or DXA methods to indirect response the effect of dorzagliatin on the liver steatosis or hepatic triglyceride content in the future.

It would be remiss not to point out the limitations of this study. The sample size of this study was initially small. Then, we selected a population of drug-naïve patients with T2DM who might be in the early stages of diabetes. This may mean that our conclusions are not generalizable to patients with severe T2DM, such that further clinical trials are needed. Finally, no comparisons with other hypoglycemic drugs were designed or obtained, and we provided no continued follow-up after drug discontinuation in this study, limitations that must be considered and preferably eliminated in future trials.

## 5. Conclusion

The present study has demonstrated that dorzagliatin monotherapy can be effective, safe, and well tolerated. We show that, in this study, dorzagliatin treatment not only improved glycemic control but also effectively reduced glycemic fluctuations, an effect most obviously observed in postprandial glucose fluctuations. More studies are required to clarify the effects of dorzagliatin on poorly controlled patients in combination with other hypoglycemic drugs and on cardiovascular and renal outcomes of patients susceptible to T2DM in the future.

## Figures and Tables

**Table 1:** Clinical characteristics of the patients before and after intervention.

	Week 0	Week 52	*P* value
Weight (kg)	69.23 ± 9.69	69.13 ± 9.62	0.816
BMI (kg/m^2^)	25.50 ± 2.14	25.46 ± 1.91	0.825
SBP (mmHg)	126.16 ± 12.51	130.25 ± 11.32	0.138
DBP (mmHg)	79.40 ± 8.25	82.08 ± 8.15	0.132
Hemoglobin (g/L)	140.62 ± 28.46	146.05 ± 11.88	0.405
ALT (U/L)	18.32 ± 6.26	21.52 ± 10.02	0.027
AST (U/L)	17.32 ± 2.91	29.96 ± 5.44	0.012
Creatinine (*μ*mol/L)	67.64 ± 11.59	69.36 ± 14.89	0.359
eGFR (mL/min/1.73 m^2^)	98.71 ± 22.89	96.05 ± 21.27	0.351
Total cholesterol (mmol/L)	4.68 ± 0.98	5.05 ± 1.14	0.028
HDL-C (mmol/L)	1.16 ± 0.24	1.26 ± 0.27	0.008
LDL-C (mmol/L)	2.72 ± 0.79	2.93 ± 1.00	0.234
Triglycerides (mmol/L)	1.82 ± 1.37	2.04 ± 1.33	0.046

BMI: body mass index; SBP: systolic blood pressure; DBP: diastolic blood pressure; ALT: alanine aminotransferase; AST: aspartate transaminase; eGFR: estimated glomerular filtration rate; HDL-C: high-density lipoprotein cholesterol; LDL-C: low-density lipoprotein cholesterol. All data are shown as mean ± SD.

**Table 2:** Glucose fluctuation parameters of the patients before and after intervention.

	Before	After	*P* value
MBG (mmol/L)	10.21 ± 2.07	8.52 ± 1.75	0.002
SDBG (mmol/L)	2.00 ± 0.71	1.46 ± 0.77	0.006
MAGE (mmol/L)	5.18 ± 1.92	3.65 ± 1.73	0.003
LAGE (mmol/L)	7.96 ± 2.65	5.98 ± 2.42	0.008
ADRR	1.63 ± 0.36	1.16 ± 0.43	<0.001
LBGI	0 (0, 0.12)	0.07 (0, 0.99)	0.091
HBGI	9.08 (4.81, 15.09)	4.66 (1.61, 8.47)	0.002
TIR (3.9−10 mmol/L, %)	66.84 (22.40, 80.90)	89.58 (58.07, 98.96)	0.005
TAR (>10 mmol/L, %)	33.16 (19.10, 77.60)	9.72 (0, 41.93)	0.004
TBR (<3.9 mmol/L, %)	0 (0, 0)	0 (0, 0)	0.180

MBG: mean blood glucose (mmol/L); SDBG: standard deviation of mean blood glucose (mmol/L); MAGE: mean amplitude of glycemic excursions (mmol/L); LAGE: largest amplitude of glycemic excursions (mmol/L); ADRR: average daily risk range; LBGI: low blood glucose index; HGBI: high blood glucose index; TIR: time in range: percentage of time in the range of 3.9–10 mmol/L; TAR: time above range: percentage of time >10 mmol/L; TBR: time below range: percentage of time <3.9 mmol/L. Continuous variables with normal distribution were presented as mean ± SD; non-normal variables are shown as median (interquartile range).

**Table 3:** Adverse events and hypoglycemic events during the 52-week treatment period.

	No. of patients
SAEs	0
Any AE	5
Upper respiratory tract infection	2
Hypertension	0
Hyperlipidemia	1
Abnormal hepatic function	0
Severe hypoglycemia	0
Clinically significant hypoglycemia (blood glucose level <54 mg·dl^−1^)	2

SAE: serious adverse event; AE: adverse event.

## Data Availability

The datasets used to support the findings of this study are available from the corresponding authors on reasonable request.

## References

[1] Tahrani A. A., Barnett A. H., Bailey C. J. (2016). Pharmacology and therapeutic implications of current drugs for type 2 diabetes mellitus. *Nature Reviews Endocrinology*.

[2] Upadhyay J., Polyzos S. A., Perakakis N. (2018). Pharmacotherapy of type 2 diabetes: an update. *Metabolism*.

[3] Banegas J. R., López-García E., Dallongeville J. (2011). Achievement of treatment goals for primary prevention of cardiovascular disease in clinical practice across Europe: the EURIKA study. *European Heart Journal*.

[4] Khunti K., Ceriello A., Cos X., De Block C. (2018). Achievement of guideline targets for blood pressure, lipid, and glycaemic control in type 2 diabetes: a meta-analysis. *Diabetes Research and Clinical Practice*.

[5] Toulis K. A., Hanif W., Saravanan P. (2017). All-cause mortality in patients with diabetes under glucagon-like peptide-1 agonists: a population-based, open cohort study. *Diabetes & Metabolism*.

[6] Toulis K. A., Willis B. H., Marshall T. (2017). All-cause mortality in patients with diabetes under treatment with dapagliflozin: a population-based, open-cohort study in the health improvement network database. *Journal of Clinical Endocrinology & Metabolism*.

[7] Tahrani A. A., Bailey C. J., Del Prato S., Barnett A. H. (2011). Management of type 2 diabetes: new and future developments in treatment. *The Lancet*.

[8] Petersen M. C., Vatner D. F., Shulman G. I. (2017). Regulation of hepatic glucose metabolism in health and disease. *Nature Reviews Endocrinology*.

[9] Sternisha S. M., Miller B. G. (2019). Molecular and cellular regulation of human glucokinase. *Archives of Biochemistry and Biophysics*.

[10] Matschinsky F. M., Wilson D. F. (2019). The central role of glucokinase in glucose homeostasis: a perspective 50 Years after demonstrating the presence of the enzyme in islets of langerhans. *Frontiers in Physiology*.

[11] Gomis R. R., Favre C., García-Rocha M., Fernández-Novell J. M., Ferrer J. C., Guinovart J. J. (2003). Glucose 6-phosphate produced by gluconeogenesis and by glucokinase is equally effective in activating hepatic glycogen synthase. *Journal of Biological Chemistry*.

[12] Toulis K. A., Nirantharakumar K., Pourzitaki C., Barnett A. H., Tahrani A. A. (2020). Glucokinase activators for type 2 diabetes: challenges and future developments. *Drugs*.

[13] Velho G., Blanché H., Vaxillaire M. (1997). Identification of 14 new glucokinase mutations and description of the clinical profile of 42 MODY-2 families. *Diabetologia*.

[14] Ellard S., Beards F., Allen L. I. (2000). A high prevalence of glucokinase mutations in gestational diabetic subjects selected by clinical criteria. *Diabetologia*.

[15] Njølstad P. R., Sagen J. V., Bjørkhaug L. (2003). Permanent neonatal diabetes caused by glucokinase deficiency: inborn error of the glucose-insulin signaling pathway. *Diabetes*.

[16] Nakamura A., Omori K., Terauchi Y. (2021). Glucokinase activation or inactivation: which will lead to the treatment of type 2 diabetes. *Diabetes, Obesity and Metabolism*.

[17] Zhu D., Gan S., Liu Y. (2018). Dorzagliatin monotherapy in Chinese patients with type 2 diabetes: a dose-ranging, randomised, double-blind, placebo-controlled, phase 2 study. *Lancet Diabetes & Endocrinology*.

[18] Xu H., Sheng L., Chen W. (2016). Safety, tolerability, pharmacokinetics, and pharmacodynamics of novel glucokinase activator HMS5552: results from a first-in-human single ascending dose study. *Drug Design, Development and Therapy*.

[19] Šoupal J., Škrha J., Fajmon M. (2014). Glycemic variability is higher in type 1 diabetes patients with microvascular complications irrespective of glycemic control. *Diabetes Technology & Therapeutics*.

[20] Kwai N. C., Arnold R., Poynten A. M., Krishnan A. V. (2016). Association between glycemic variability and peripheral nerve dysfunction in type 1 diabetes. *Muscle & Nerve*.

[21] Costantino S., Paneni F., Battista R. (2017). Impact of glycemic variability on chromatin remodeling, oxidative stress, and endothelial dysfunction in patients with type 2 diabetes and with target HbA(1c) levels. *Diabetes*.

[22] Matthews D. R., Hosker J. P., Rudenski A. S., Naylor B. A., Treacher D. F., Turner R. C. (1985). Homeostasis model assessment: insulin resistance and beta-cell function from fasting plasma glucose and insulin concentrations in man. *Diabetologia*.

[23] Wang Y., Li H., Gao H. (2022). Effect of chiglitazar and sitagliptin on glucose variations, insulin resistance and inflammatory-related biomarkers in untreated patients with type 2 diabetes. *Diabetes Research and Clinical Practice*.

[24] Phillips D. I., Clark P. M., Hales C. N., Osmond C. (1994). Understanding oral glucose tolerance: comparison of glucose or insulin measurements during the oral glucose tolerance test with specific measurements of insulin resistance and insulin secretion. *Diabetic Medicine*.

[25] Haffner S. M., Miettinen H., Gaskill S. P., Stern M. P. (1995). Decreased insulin secretion and increased insulin resistance are independently related to the 7-year risk of NIDDM in Mexican-Americans. *Diabetes*.

[26] Caro J. F., Triester S., Patel V. K., Tapscott E. B., Frazier N. L., Dohm G. L. (1995). Liver glucokinase: decreased activity in patients with type II diabetes. *Hormone and Metabolic Research*.

[27] Grimsby J., Sarabu R., Corbett W. L. (2003). Allosteric activators of glucokinase: potential role in diabetes therapy. *Science*.

[28] Meininger G. E., Scott R., Alba M. (2011). Effects of MK-0941, a novel glucokinase activator, on glycemic control in insulin-treated patients with type 2 diabetes. *Diabetes Care*.

[29] Sarabu R., Bizzarro F. T., Corbett W. L. (2012). Discovery of piragliatin--first glucokinase activator studied in type 2 diabetic patients. *Journal of Medicinal Chemistry*.

[30] Kiyosue A., Hayashi N., Komori H., Leonsson-Zachrisson M., Johnsson E. (2013). Dose-ranging study with the glucokinase activator AZD1656 as monotherapy in Japanese patients with type 2 diabetes mellitus. *Diabetes, Obesity and Metabolism*.

[31] Erion D. M., Lapworth A., Amor P. A. (2014). The hepatoselective glucokinase activator PF-04991532 ameliorates hyperglycemia without causing hepatic steatosis in diabetic rats. *PLoS One*.

[32] Sharma S., Wadhwa K., Choudhary M., Budhwar V. (2021). Ethnopharmacological perspectives of glucokinase activators in the treatment of diabetes mellitus. *Natural Product Research*.

[33] Kovatchev B., Cobelli C. (2016). Glucose variability: timing, risk analysis, and relationship to hypoglycemia in diabetes. *Diabetes Care*.

[34] Pazos-Couselo M., García-López J. M., González-Rodríguez M. (2015). High incidence of hypoglycemia in stable insulin-treated type 2 diabetes mellitus: continuous glucose monitoring vs. self-monitored blood glucose. Observational prospective study. *Canadian Journal of Diabetes*.

[35] Mazze R., Strock E., Morgan B., Wesley D., Bergenstal R., Cuddihy R. (2009). Diurnal glucose patterns of exenatide once weekly: a 1-year study using continuous glucose monitoring with ambulatory glucose profile analysis. *Endocrine Practice*.

[36] Irace C., Fiorentino R., Carallo C., Scavelli F., Gnasso A. (2011). Exenatide improves glycemic variability assessed by continuous glucose monitoring in subjects with type 2 diabetes. *Diabetes Technology & Therapeutics*.

[37] Kim H. S., Shin J. A., Lee S. H. (2013). A comparative study of the effects of a dipeptidyl peptidase-IV inhibitor and sulfonylurea on glucose variability in patients with type 2 diabetes with inadequate glycemic control on metformin. *Diabetes Technology & Therapeutics*.

[38] Henry R. R., Strange P., Zhou R. (2018). Effects of dapagliflozin on 24-hour glycemic control in patients with type 2 diabetes: a randomized controlled trial. *Diabetes Technology & Therapeutics*.

[39] Kota S., Satya Krishna S., Modi K. D. (2013). Glycemic variability: clinical implications. *Indian Journal of Endocrinology and Metabolism*.

[40] Yu P. C., Bosnyak Z., Ceriello A. (2010). The importance of glycated haemoglobin (HbA(1c)) and postprandial glucose (PPG) control on cardiovascular outcomes in patients with type 2 diabetes. *Diabetes Research and Clinical Practice*.

[41] Meigs J. B., Nathan D. M., D’Agostino R. B., Wilson P. W. (2002). Fasting and postchallenge glycemia and cardiovascular disease risk: the Framingham Offspring Study. *Diabetes Care*.

[42] Hanson R. L., Pratley R. E., Bogardus C. (2000). Evaluation of simple indices of insulin sensitivity and insulin secretion for use in epidemioiogic studies. *American Journal of Epidemiology*.

[43] Kawata S., Nakamura A., Miyoshi H. (2022). Glucokinase activation leads to an unsustained hypoglycaemic effect with hepatic triglyceride accumulation in db/db mice. *Diabetes, Obesity and Metabolism*.

